# Consensus-based perspectives on integrating IPX203 into Parkinson’s disease management

**DOI:** 10.1007/s00415-026-14186-1

**Published:** 2026-10-11

**Authors:** Günter Höglinger, Carsten Buhmann, Georg Ebersbach, Margherita Fabbri, Georg Kägi, Javier Pagonabarraga, Diego Santos García, Alessandro Tessitore, Michele Tinazzi

**Affiliations:** 1 Department of Neurology, LMU University Hospital, Ludwig-Maximilians-Universität (LMU) München, Munich, Germany; 2 German Center for Neurodegenerative Diseases (DZNE), Munich, Germany; 3 Munich Cluster for Systems Neurology (SyNergy), Munich, Germany; 4 Department of Neurology, University Medical Center Hamburg-Eppendorf, Hamburg, Germany; 5 Movement Disorder Hospital, Beelitz-Heilstätten, Germany; 6 Clinical Investigation Center CIC1436, Department of Clinical Pharmacology and Neurosciences, Parkinson Expert Centre and NeuroToul Center of Excellence in Neurodegeneration (COEN) of Toulouse, INSERM, University of Toulouse, CHU of Toulouse, Toulouse, France; 7 Department of Neurology, Inselspital, Bern University Hospital, University of Bern, Bern, Switzerland; 8 Movement Disorder Unit, Neurology Department, Hospital de La Santa Creu I Sant Pau, Barcelona, Spain; 9 Department of Medicine, Autonomous University of Barcelona, Barcelona, Spain; 10 Sant Pau Biomedical Research Institute (IIB-Sant Pau), Barcelona, Spain; 11 Centro de Investigación en Red, Enfermedades Neurodegenerativas (CIBERNED), Madrid, Spain; 12 Complejo Hospitalario Universitario de A Coruña (CHUAC), A Coruña, Spain; 13 Grupo de Investigación en Enfermedad de Parkinson y Otros Trastornos del Movimiento, INIBIC (Instituto de Investigación Biomédica de A Coruña), A Coruña, Spain; 14 Hospital San Rafael, A Coruña, Spain; 15 Fundación Degen, A Coruña, Spain; 16 Department of Advanced Medical and Surgical Sciences, University of Campania, “L. Vanvitelli”, Naples, Italy; 17 Department of Neuroscience, Biomedicine and Movement, University of Verona, Verona, Italy

**Keywords:** Clinical consensus, IPX203, Levodopa, Modified-release formulation, Motor fluctuation, Parkinson disease

## Abstract

Motor fluctuations are a common and burdensome complication of Parkinson’s disease (PD) when the disease progresses, largely driven by the short half-life and variable absorption of immediate-release (IR) levodopa. IPX203 is a next-generation modified-release levodopa/carbidopa formulation that delivers rapid onset and sustained plasma levodopa concentrations through IR granules and extended-release pellets. Clinical studies have shown that IPX203 increases Good ON time (ON time without dyskinesia plus ON time with non-troublesome dyskinesia, equivalent to ON time without troublesome dyskinesia), reduces OFF time and improves motor symptom control compared with IR levodopa/carbidopa. In anticipation of the introduction of IPX203 in Europe, nine PD specialists convened to develop consensus recommendations for its use in clinical practice. Through a structured process incorporating predefined statements, voting and moderated discussion, the group reached high levels of agreement (88.9% to 100%) across seven topics. These included defining motor symptoms control, identifying patients most likely to benefit from IPX203, positioning of IPX203 within the treatment pathway, practical considerations for switching to IPX203 from current regimens and recommendations for monitoring and follow-up. The experts emphasised that IPX203 should be considered for patients with motor fluctuations insufficiently controlled on optimised oral levodopa therapy, before introduction of device-aided therapies. They highlighted the need for flexible dose adjustments, comprehensive patient and neurologist education and practical tools to support conversion to IPX203. This consensus insight provides practical guidance for integrating IPX203 into routine care of PD patients with motor fluctuations, aiming to increase mobility and reduce treatment burden for patients with PD.

**Supplementary Information:**

The online version contains supplementary material available at https://doi.org/10.1007/s00415-026-14186-1.

## Introduction

Levodopa remains the primary foundational treatment for managing motor symptoms in Parkinson’s disease (PD) [1, 2]. Levodopa is typically combined with an inhibitor of dopa decarboxylase (DDC), such as carbidopa or benserazide [3–5]. In patients with advanced PD, frequent dosing with an immediate-release (IR) levodopa formulation is often required to maintain adequate symptom control [5, 6]. However, the pharmacokinetic limitations of IR levodopa/DDC combinations, such as short half-life and variable absorption, result in inconsistent plasma levodopa concentrations [3]. Fluctuations in plasma levodopa concentrations are common and can emerge within 2 years of starting therapy [7–9]. They represent a major, though not exclusive, contributor to the development of motor complications, including delayed ON periods, unpredictable ON–OFF motor fluctuations, end-of-dose wearing OFF and peak-dose dyskinesias, as well as non-motor symptoms that negatively impact quality of life. To maintain PD symptom control, patients often need increasingly frequent levodopa/DDC dosing, adjunctive medications and may eventually require device-aided therapies (DATs), such as deep brain stimulation (DBS) and or infusion therapies/pumps [10–12]. For appropriately selected patients, DATs can provide substantial benefits in managing motor fluctuations and other symptoms of advanced PD; however, these interventions are invasive, complex and require close clinical supervision [12]. Real-world data suggest that optimised levodopa dosing and appropriate adjunctive therapies can help stabilise long-standing PD and maintain a good quality of life [13, 14]. Nevertheless, many patients continue on oral therapies over the long term [15], including a substantial proportion of patients with advanced PD who are considered eligible for DATs but have not yet undergone these interventions [16]. In one real-world study, nearly 40% of DAT-eligible patients had no DAT planned, with the need for additional time to make a treatment decision and patient refusal among the most commonly reported reasons [16]. Consequently, timely and tailored treatment with an appropriate levodopa/DDC formulation, potentially combined with adjunctive therapies, may help optimise symptom control and support a more individualised approach to treatment planning, including the timing of DAT initiation, while also providing an alternative for patients who are not willing or are unsuitable to undergo invasive procedures.

IPX203, a next-generation modified-release (MR) levodopa/carbidopa formulation, was developed to overcome the limitations of existing levodopa formulations [17]. Once initiated, IPX203 may improve motor fluctuations and quality of life [17, 18]. By combining IR granules with extended-release (ER) pellets in a single capsule, IPX203 was engineered to optimise levodopa delivery and absorption, providing both rapid onset and sustained plasma levodopa concentrations [19]. The formulation provides 100% of the carbidopa dose and 25% of the levodopa dose via IR granules, while the remaining 75% of levodopa is gradually released from ER pellets engineered with sustained release, mucoadhesive and enteric coatings to enhance absorption in the proximal small intestine [19]. This approach aims to reduce peak-to-trough variability and dosing frequency [20]. Clinical studies in patients with PD have demonstrated that IPX203 increases Good ON time (ON time without dyskinesia plus ON time with non-troublesome dyskinesia, equivalent to ON time without troublesome dyskinesia), significantly reduces OFF time and improves motor symptom control compared with IR levodopa/carbidopa [3, 17, 21, 22]. Following approval by the United States Food and Drug Administration in 2024 [23], IPX203 (Hopledo) was subsequently approved for use in Europe in August 2026 [24].

In preparation for its introduction, a board of European expert neurologists was convened to refine PD management strategies for the use of IPX203. The collaborative forum aimed to build strategic alignment on clinical definitions of motor fluctuations, optimise treatment pathways for IPX203 and co-develop recommendations to support its broad implementation in routine practice. This article provides an overview of important themes in the management of PD with IPX203 that emerged from these discussions.

### Expert board process and consensus development

A scientific board comprising nine European experts (the authors of this manuscript) convened in person in September 2025 to develop consensus on the management of PD in anticipation of the availability of IPX203. Experts were selected by the study sponsor (Zambon Biotech SA) based on recognised expertise in Parkinson’s disease management and active involvement in clinical research, education or guideline development. An initial pool of clinicians from multiple European countries was identified to ensure broad representation of clinical practice across Europe. Participation was voluntary, and the final expert board comprised those who accepted the invitation and were available to contribute within the project timelines. The nine participating experts represented Germany, Italy, Spain, France and Switzerland. The participants had extensive clinical experience (18–36 years) and specialised expertise in the management of PD, providing diagnostic and therapeutic services, including oral treatment and DATs.

The expert board focused on the following five core objectives: (1) establishing a clear and shared definition of motor fluctuations in PD; (2) defining clinical criteria and decision-making pathways for initiating treatment with IPX203; (3) developing strategies for optimising treatment when transitioning from current PD therapies to IPX203; (4) identifying key considerations for creating practical tools to support conversion to IPX203; and (5) designing strategies for effective knowledge dissemination to general neurologists to help ensure that IPX203 reaches eligible patients.

Consensus building followed a structured and interactive format designed to facilitate both individual reflection and collective alignment. The sponsor developed an initial set of eight draft statements, which were reviewed with the working group coordinator (GH) and subsequently presented to the expert board for discussion. These statements addressed key aspects of PD management and the potential integration of IPX203, and were developed based on up-to-date literature and informed by current unmet needs of patients with PD.

Expert board participants reviewed the predefined statements and rated their agreement with each statement using a three-point scale: ‘disagree’, ‘neutral’ or ‘agree’. Following the initial round of voting, the level of agreement for each statement was calculated and presented back to the group. These results informed a moderated roundtable discussion aimed at exploring differing viewpoints, clarifying interpretations and refining the statements where necessary. During the discussion, one of the initial eight statements was deemed outside of the scope of the initiative because it was not centred around the role of IPX203 and was therefore not progressed to a second round of voting. The revised statements were then submitted to a second round of voting, allowing the group to validate the updated statements and confirm alignment. Key insights and conclusions from both the voting process and discussions were captured in real time to ensure accurate representation of the collective recommendations.

### Consensus building on the use of IPX203 for the management of PD

**Fig. 1:**
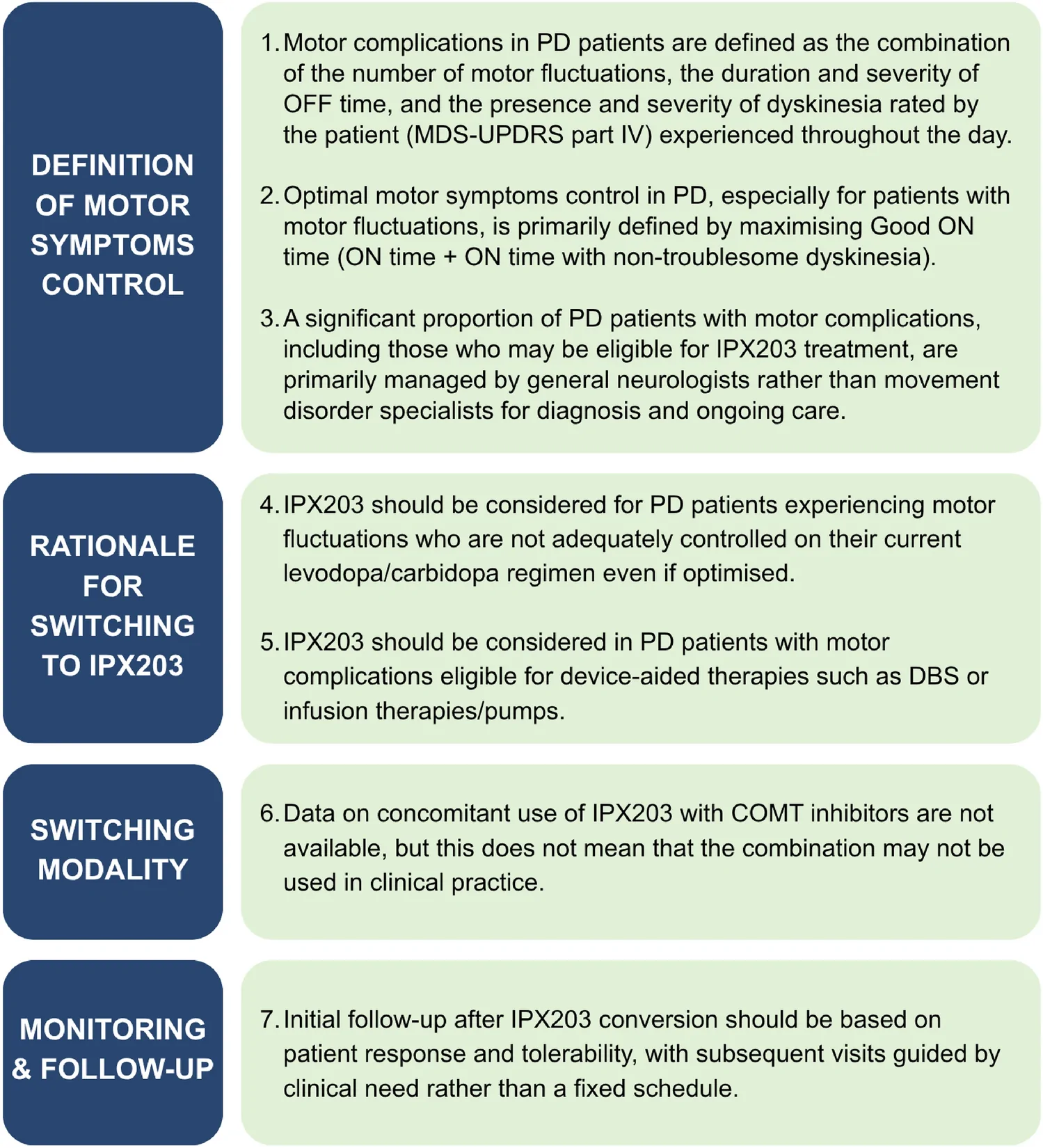
Consensus on IPX203 management. *COMT* catechol-O-methyltransferase, *DBS* deep brain stimulation, *MDS-UPDRS* Movement Disorder Society-Unified Parkinson’s Disease Rating Scale, *PD* Parkinson’s disease

All nine expert participants completed the voting process. They voted on the predefined consensus statements that described the definition of motor symptoms control, rationale for switching to IPX203, switching modality and recommendations for monitoring and follow-up (Fig. 1). Following refinement of the statements, the level of agreement ranged from 88.9% to 100%. The final statements are described in the following sections.

### Definition of motor symptoms control

#### Statement 1: Motor complications in PD patients are defined as the combination of the number of motor fluctuations, the duration and severity of OFF time, and the presence and severity of dyskinesia rated by the patient (MDS-UPDRS part IV) experienced throughout the day (100% agreement)

Participants emphasised the importance of clearly differentiating motor fluctuations from dyskinesia. They noted that the two terms should not be grouped together indiscriminately, without a clear differentiation of each concept. There was a strong opinion that the definition should explicitly include motor complications as a broader term, encompassing both dyskinesia and motor fluctuations.

Concerns were also raised regarding the exclusion of non-motor symptoms from the definition of fluctuations. Non-motor fluctuations (e.g. anxiety, pain, cognitive issues) are highly relevant to patients and correlated with their overall experience of motor fluctuations. Participants further stressed the need to clarify how “severity” is defined and measured, given that “troublesomeness” is a patient-reported concept that may not correlate directly with clinical severity. Even mild dyskinesia can occasionally be perceived as troublesome by some patients.

The role of existing assessment tools and objective parameters was another point of discussion. Some participants questioned the reliance on the patient-reported Hauser diary alone [25]. The participants also identified a clear need for objective assessment of functional parameters, along with a standardised severity rating scale that incorporates both patient and clinician perspectives. The Movement Disorder Society-Unified Parkinson’s Disease Rating Scale (MDS-UPDRS) part IV – motor complications [26], which combines patient-reported information with clinical judgment, is a validated and universally applicable tool for broader implementation in both clinical trials and routine practice.

Finally, perspectives differed on the desired complexity of the definition. Some participants advocated for a simpler, more straightforward definition of motor fluctuations (e.g. any change in motor behaviour related to dopaminergic treatment), whereas others argued for a more comprehensive definition that captures the multifaceted nature of the phenomenon.

#### Statement 2: Optimal motor symptoms control in PD, especially for patients with motor fluctuations, is primarily defined by maximising Good ON time (ON time without dyskinesia + ON time with non-troublesome dyskinesia, equivalent to ON time without troublesome dyskinesia) (100% agreement)

Participants highlighted the need for clearer communication regarding the definition of the primary endpoint in the pivotal, randomised, phase 3 RISE-PD trial of IPX203 versus IR levodopa/carbidopa: Good ON time (i.e. the sum of ON time without dyskinesia and ON time with non-troublesome dyskinesia, equivalent to ON time without troublesome dyskinesia) [21]. They noted that achieving ON time without any dyskinesia is rarely achievable with levodopa treatment, raising concerns about the practicality of the current endpoint definition.

Additionally, the exclusion of freezing of gait from the overall functional assessment of optimal symptom control was considered problematic, as freezing of gait can occur frequently during ON periods. Overall, freezing of gait affects ~38–65% of patients with PD, depending on disease duration [27]. Its omission may result in an incomplete evaluation of motor function and treatment efficacy, potentially limiting the accuracy of outcome measures.

#### Statement 3: A significant proportion of PD patients with motor complications, including those who may be eligible for IPX203 treatment, are primarily managed by general neurologists rather than movement disorder specialists for diagnosis and ongoing care (100% agreement)

Participants noted that the management of patients with PD varies significantly across Europe due to geographic heterogeneity and differences in local healthcare structures. In some regions, even patients with advanced disease are primarily managed by general neurologists, a trend often driven by the limited capacity of specialist centres. Where access to movement disorder specialists is limited, general neurologists often manage patients with early-stage PD, as well as those with more complex, advanced-stage disease.

Physician involvement typically correlates with disease stage: patients with early or mild PD are more commonly managed by general neurologists, while advanced stage patients ideally require movement disorder specialist involvement, particularly when they present with complex motor complications. Participants noted that referral to a movement disorder specialist should be considered when patients begin to experience motor fluctuations. In this context, participants emphasised that IPX203 eligibility is currently often associated with patients experiencing moderate to severe motor fluctuations, rather than early or mild fluctuations.

Although not included in the IPX203 product label [23], the RISE-PD trial applied ≥ 2.5 h of OFF time per day as a key inclusion criterion [21]. Participants agreed that this threshold could serve as a practical benchmark for identifying patients who may benefit significantly from specialist evaluation and potential treatment with IPX203. Additionally, the “5–2-1” rule (levodopa taken ≥ 5 times per day, ≥ 2 h of OFF time daily, and ≥ 1 h of troublesome dyskinesia) may also help to identify such patients [28].

### Rationale for switching to IPX203

#### Statement 4: IPX203 should be considered for PD patients experiencing motor fluctuations who are not adequately controlled on their current levodopa/carbidopa regimen even if optimised (100% agreement)

Participants expressed interest in considering IPX203 for a wider range of patients, i.e. those experiencing any motor fluctuations, rather than only those with moderate to severe motor fluctuations. They emphasised that patient burden, particularly the subjective experience of fluctuations, plays a critical role in treatment decisions. Even when fluctuations are not judged to be clinically severe, they can still be highly troublesome for patients. In such cases, the benefits of IPX203, such as reduced dosing frequency and sustained ON time, may be valuable across the full spectrum of motor fluctuations.

The group also discussed the need to clarify whether the term “troublesome”, defined as fluctuations that negatively impact patients’ quality of life, well-being and autonomy, should be explicitly included in the statement to reflect this patient-centric perspective, or whether this concept is already implied by the phrase “not adequately controlled”. Additionally, participants clarified that the current term “levodopa/carbidopa regimen” encompasses combination therapies, as patients are often treated with multiple concomitant drugs (i.e. monoamine oxidase-B inhibitors, catechol O-methyltransferase [COMT] inhibitors, dopamine agonists and amantadine).

Importantly, the discussion highlighted that IPX203 should be viewed not merely as a later-line therapy reserved for patients who have exhausted all other oral therapies. Rather, it should be considered as a viable and earlier treatment in the optimisation process, that could serve as a first-line choice for managing motor fluctuations (which can develop within 2 years [8]) following initial levodopa optimisation.

Finally, while there was enthusiasm for broader use of IPX203, participants acknowledged that the RISE-PD trial focused on advanced disease populations [21], underscoring the need for real-world evidence or further studies to support wider application of IPX203.

#### Statement 5: IPX203 should be considered in PD patients with motor complications eligible for DATs such as DBS or infusion therapies/pumps (88.9% agreement)

Participants noted that while the concept is clinically relevant, the current evidence base is not yet strong enough to determine whether IPX203 can meaningfully delay the need for, or eligibility for, DATs. Longer‑term outcomes from the 9‑month RISE‑PD open‑label extension [22] provide encouraging signals, but the dataset remains insufficiently mature to draw firm conclusions regarding the effect of IPX203 on the progression toward DATs. Further long-term, prospective or real-world evidence are required.

Participants emphasised that IPX203 should be positioned as an option within the therapeutic sequence rather than a mandatory step intended to postpone other interventions, ensuring patient choice and shared decision-making through comprehensive discussion of all available treatment options.

The participants also highlighted the ambiguity of the term “advanced PD”, which is often associated with severe cognitive impairment or psychosis and may not accurately reflect the intended target population for IPX203. Instead, the term “Person with PD with motor complications” was seen as more appropriate, as it aligns with the core issue IPX203 is designed to address.

Participants further clarified the role of IPX203 as an oral optimisation strategy that can be used prior to DATs (DBS, magnetic resonance-guided focused ultrasound, pump-based therapies). IPX203 may offer a solution for patients who have difficulty achieving adequate control with conventional oral medications but are not yet ready or indicated for surgical or infusion-based approaches.

Finally, the discussion acknowledged the practical challenges associated with DATs, including long waiting lists for some procedures (e.g. DBS), and the importance of ensuring that patients are clinically ready for more invasive procedures.

### Switching modality

#### Statement 6: Data on concomitant use of IPX203 with COMT inhibitors are not available, but this does not mean that the combination may not be used in clinical practice (100% agreement)

Participants acknowledged a critical evidence gap regarding the concomitant use of IPX203 with COMT inhibitors. A strategy to enhance levodopa availability is to limit its conversion into the inactive metabolite 3-O-methyldopa by inhibiting the enzyme COMT. Because COMT inhibition alters levodopa metabolism and increases its bioavailability, allowing these drugs in RISE-PD would have introduced variability in levodopa exposure and added confounding factors. For this reason, COMT inhibitors were excluded to isolate the specific effect of IPX203 on motor fluctuations (ON time and OFF time). This exclusion does not imply that IPX203 cannot be combined with COMT inhibitors in clinical practice; however, no safety or efficacy data on this combination are currently available [18].

Despite this, participants emphasised that real-world practice will inevitably include patients receiving COMT inhibitors [29] who may also be candidates for IPX203. This underscores the need for further research, such as phase 4 trials or real-world evidence, to evaluate these combinations, provide pharmacokinetic data and guide clinicians in managing potential risks such as dyskinesia.

Concerns were raised regarding potential pharmacokinetic interactions when combining COMT inhibitors and IPX203. COMT inhibitors increase levodopa bioavailability and prolong its half-life [30]. When combined with the sustained-release properties of IPX203, this could lead to potentially higher peak plasma concentrations of levodopa in certain cases (e.g. when used in combination with opicapone [31]) compared with IPX203 monotherapy, thereby increasing the risk and severity of dyskinesia. Participants noted that this represents a significant safety concern that requires careful monitoring.

The group also stressed the importance of clear clinical guidance on dosing adjustments for COMT inhibitors when used with IPX203, given differences in dosing frequency. IPX203 is typically administered 2–4 times daily [23], whereas COMT inhibitor regimens might involve additional dosing [32]. Without management strategies such as levodopa dose reduction, this discrepancy could significantly influence dyskinesia risk, as higher frequency and doses of COMT inhibitors have been associated with an increased incidence of dyskinesia, creating a complex challenge for optimising combined regimens [33].

### Monitoring and follow-up

#### Statement 7: Initial follow-up after IPX203 conversion should be based on patient response and tolerability, with subsequent visits guided by clinical need rather than a fixed schedule (100% agreement)

Participants raised concerns about the practicality of scheduling follow-up visits within the first few days after switching to IPX203. Although some recommendations suggest follow-up within 1–3 days of conversion [34], the group noted that this approach is often unfeasible in routine clinical practice due to substantial clinical burden and resource constraints in busy practices. Instead, participants advocated for a more flexible approach, shifting toward patient-initiated contact for early concerns rather than universally pre-scheduled follow-up visits. This strategy empowers patients to report issues promptly while ensuring timely responses from the clinic.

There was strong agreement on the importance of comprehensive patient education during conversion to IPX203. Participants emphasised the value of providing clear, structured information on expected adverse events (AEs) and symptoms, guidance on identifying problems that require clinic contact, managing overdosing and increased fluctuations, and instructions on actions such as tapering or re-titration if symptoms arise within the first 1–2 weeks. Participants emphasised the need to develop effective, easy-to-understand educational materials that manage expectations and support self-management while avoiding unnecessary anxiety.

Finally, participants highlighted the necessity of standardised follow-up guidelines in real-world practice, supported by efficient communication channels (e.g. phone or digital platforms) and trained support staff to manage patient queries and provide initial advice, thereby reducing the direct burden on physicians for minor issues.

### IPX203 positioning, future research and evidence needs

Participants recognised IPX203 as a useful treatment option for patients with PD experiencing motor fluctuations. Discussion focused on how the combined IR and ER levodopa/carbidopa formulation, together with the potential for less frequent dosing, may reduce treatment burden and improve adherence. Attention was given to identifying the patient populations most likely to benefit from IPX203 and how it may be positioned within existing treatment pathways. Participants noted the need for additional clinical and real-world data to better understand the effects of IPX203.

Several important evidence gaps emerged during the discussions that may inform future clinical research and the gathering of real-world evidence. Among these, the potential benefits of IPX203 in patients at earlier stages of disease who are already experiencing motor fluctuations warrants further investigation. The potential for IPX203 to prevent the development of motor fluctuations by impacting processes at the synaptic level is an interesting future aspect to study. It also remains unknown how the higher dose and less pulsatile pharmacokinetic profile of IPX203 may affect long-term dyskinesia outcomes. Further research is also needed to better understand the long-term effects of IPX203 on non-motor symptoms including pain, sleep disorders and neuropsychiatric manifestations such as hallucinations, confusion and psychosis, particularly in patients experiencing cognitive decline.

Further work is required to better identify patients who are “*best levodopa absorbers*” versus “*poor absorbers*” (i.e. patients receiving high doses of IR levodopa at high dosing frequency but with inadequate response) [35], as this distinction influences dosing strategies and potential for overdosing. Although, preliminary data suggest the absence of a significant food interaction [23, 34], allowing IPX203 to be taken with or without food [24], a potential advantage relative to other levodopa treatments that are affected by food, further research is also needed to better understand the effects of food on IPX203 absorption and clinical response.

A clearer understanding of how IPX203 (administered 2–4 times daily) may improve night-time akinesia and early morning ON status is required. Evidence on the effects of IPX203 on orthostatic hypotension and freezing of gait would also be valuable. Differentiating between mild, moderate and severe fluctuations based on objective and subjective measures (e.g. OFF time between doses, patient’s description of "troublesome", eventually wearable sensors) remains an open discussion point.

Finally, further insights are needed to determine the place of IPX203 within the PD treatment algorithm, including how it compares with other drug classes (e.g. long-term dopamine agonists) and its potential to delay the need for DATs.

### Communication and education

Effective communication efforts should target both patients and general neurologists. *“Levodopa phobia”* refers to a reluctance or apprehension among both patients and clinicians regarding the optimal and timely use of levodopa in PD treatment [36]. Such concerns may be driven by misplaced fears about levodopa-induced dyskinesias at high-doses, gastrointestinal AEs and the theoretical hypothesis that levodopa could exert neurotoxic effects on dopaminergic neurons, despite a lack of evidence to support such effects. Accordingly, clinicians should clearly explain why the levodopa dose of IPX203 may appear higher than that of previous IR regimens (see Supplementary Table 1), emphasising that the formulation is designed to provide broader and more sustained levodopa exposure rather than higher peak concentrations [19]. The higher dose is intended to support efficient and prolonged delivery and should not be interpreted as an overdose. Rather, IPX203 is designed to achieve a levodopa peak concentration comparable to that of the previous IR regimen while minimising peak-to-trough fluctuations.

Beyond addressing levodopa phobia, effective communication and comprehensive patient education during conversion to IPX203 should anticipate common patient concerns and provide structured, practical guidance. Patients may feel anxious about changing a long-standing levodopa regimen, and clinicians should therefore clearly explain the rationale for conversion, including the potential benefits of more sustained symptom control and reduced dosing frequency.

Patients should be informed that IPX203 may have a slightly slower onset of effect than IR levodopa but is designed to provide more sustained symptom control throughout the day, reducing the risk that early experiences are misinterpreted as treatment failure. Clinicians should discuss the possibility of transient effects during conversion, including nausea, dizziness, dry mouth and dyskinesia, and explain that these often diminish with dose optimisation. Clear expectations regarding dosing intervals are particularly important. The MR properties of IPX203 should be emphasised, together with the importance of adhering to prescribed dosing intervals to maximise the benefits of its formulation. Clinicians should also proactively address misconceptions about levodopa treatment, such as concerns that levodopa loses effectiveness with long-term use or that ER formulations are inherently less effective than IR therapy.

Patients should be advised that high-fat meals may delay levodopa absorption and that, for those with swallowing difficulties, the capsule contents may be sprinkled on applesauce without affecting pharmacokinetics. They should also be encouraged to monitor and report changes in OFF periods, dyskinesia and overall daily functioning during the first weeks after conversion, facilitating timely and individualised treatment adjustments. Finally, patients should receive clear guidance regarding when to contact their healthcare team, particularly in the event of persistent nausea, worsening dyskinesia, unexpected OFF episodes or psychiatric adverse effects. Structured support, including nurse-led assistance and clearly defined follow-up protocols, may help address patient concerns and facilitate the management of minor issues without requiring direct physician involvement.

In addition to patient education, education of general neurologists will be essential to support the successful integration of IPX203 into routine clinical practice. While movement disorder specialists are anticipated to be the primary drivers of IPX203 adoption due to their expertise, successful integration of IPX203 into routine clinical practice will also rely on general neurologists. Education will therefore be essential, as concerns about levodopa-related AEs (e.g. dyskinesia, impulse control disorders, dopamine dysregulation syndrome and punding) may contribute to undertreatment with levodopa-based therapies [36]. Clear communication about the unique pharmacokinetic/pharmacodynamic profile of IPX203 is crucial to address these concerns. In this context, simple, visually engaging materials, such as cartoon-based information, may help convey key concepts more effectively than complex digital tools. It should also be emphasised that IPX203 is a MR formulation distinct from traditional controlled-release formulations, which have different efficacy profiles [6, 37, 38]. Providing practical tools to support the IPX203 conversion algorithm will further facilitate adoption in routine clinical practice. Collaboration with local neurological societies should be encouraged to support disseminating knowledge and best practices.

### Clinical implementation and adoption

The key to successful adoption of IPX203 is identifying the appropriate candidates for treatment. The following action points should be addressed: (1) develop standard operating procedures for IPX203 implementation, including criteria for patient selection and follow-up; (2) consider switching patients who have not been sufficiently stabilised on a treatment regimen combining oral levodopa and a DDC inhibitor [24]; (3) consider IPX203 as an option before transitioning to DATs, where it may serve as a bridging therapy or an alternative for patients who are unwilling or unsuitable to undergo invasive procedures; (4) emphasise the importance of individualised treatment, with dose selection and treatment optimisation tailored according to patient characteristics, treatment goals and local clinical practice; and (5) carefully monitor patients with pre-existing dyskinesia or nausea, as these have been identified as potential AEs associated with IPX203 [21].

## Conclusions

The expert board confirmed the potential of IPX203 to significantly improve outcomes for patients with PD. The successful adoption of IPX203 will depend on clear and consistent communication of its unique benefits, targeted education for neurologists across specialties and implementation of practical tools that support its integration into diverse clinical settings.

## Supplementary Information

Below is the link to the electronic supplementary material.

Supplementary file1 (DOCX 41 KB)

## Data Availability

Data sharing is not applicable to this article as no datasets were generated or analysed during the current study. Details related to the voting of the statements are available from the corresponding author upon reasonable request.
