# Supplementary material for: Consensus-based perspectives on integrating IPX203 into Parkinson’s disease management
Source: J Neurol. 2026 Oct 11;273(11):656. doi: 10.1007/s00415-026-14186-1 (PMC13653810; doi:10.1007/s00415-026-14186-1)
Supplement: Supplementary file 1 [file 415_2026_14186_MOESM1_ESM.docx]

# Consensus‑based perspectives on integrating IPX203 into Parkinson’s disease management

# Supplementary materials

**Journal name:** *Journal of Neurology*

**Authors:** Günter Höglinger^1,2,3^, Carsten Buhmann^4^, Georg Ebersbach^5^, Margherita Fabbri^6^, Georg Kägi^7^, Javier Pagonabarraga^8, 9, 10, 11^, Diego Santos Garcia^11,12, 13, 14, 15^, Alessandro Tessitore^16^, Michele Tinazzi^17^

**Affiliations**

^1^ Department of Neurology, LMU University Hospital, Ludwig-Maximilians-Universität (LMU) München, Munich, Germany

^2^ German Center for Neurodegenerative Diseases (DZNE), Munich, Germany

^3^ Munich Cluster for Systems Neurology (SyNergy), Munich, Germany

^4^ Department of Neurology, University Medical Center Hamburg-Eppendorf, Hamburg, Germany

^5^ Movement Disorder Hospital, Beelitz-Heilstätten, Germany

^6^ Clinical Investigation Center CIC1436, Department of Clinical Pharmacology and Neurosciences, Parkinson Expert Centre and NeuroToul Center of Excellence in Neurodegeneration (COEN) of Toulouse; INSERM, University of Toulouse, CHU of Toulouse, Toulouse, France

^7^ Department of Neurology, Inselspital, Bern University Hospital, University of Bern, Switzerland

^8^ Movement Disorder Unit, Neurology Department, Hospital de la Santa Creu i Sant Pau, Barcelona, Spain

^9^ Department of Medicine, Autonomous University of Barcelona, Barcelona, Spain

^10^ Sant Pau Biomedical Research Institute (IIB-Sant Pau), Barcelona, Spain

^11^ Centro de Investigación en Red, Enfermedades Neurodegenerativas (CIBERNED), Madrid, Spain

^12^ Complejo Hospitalario Universitario de A Coruña (CHUAC), A Coruña, Spain

^13^ Grupo de Investigación en Enfermedad de Parkinson y otros Trastornos del Movimiento, INIBIC (Instituto de Investigación Biomédica de A Coruña), A Coruña, Spain

^14^ Hospital San Rafael, A Coruña, Spain

^15^ Fundación Degen, A Coruña, Spain

^16^ Department of Advanced Medical and Surgical Sciences, University of Campania, “L. Vanvitelli”, Naples, Italy

^17^ Department of Neuroscience, Biomedicine and Movement, University of Verona, Italy

**Corresponding author:** Prof. Dr. Günter Höglinger, MD, FEAN. Email: Guenter.Hoeglinger@med.uni-muenchen.de

# Supplementary tables

## Supplementary Table 1. Conversion from immediate-release levodopa/carbidopa to IPX203 [1].

| **Total daily IR levodopa dose (mg)** | **Most frequent IR levodopa single dose ^a^(mg)** | **Recommended starting dosage of Hopledo (IPX203) (mg of levodopa)** |
| --- | --- | --- |
| <500 mg | 100 | 280 twice daily |
|  | 150 | 420 twice daily |
|  | 200 | 560 twice daily |
| ≥500 mg | 100 | 280 three times daily |
|  | 150 | 420 three times daily |
|  | 200^b^ | 560 three times daily |
|  | >200^b^ | 700 three times daily |

The dosages of IR levodopa/carbidopa products are not interchangeable on a 1:1 basis with the dosages of IPX203 (Hopledo). After determining the patient’s total daily dosage of IR levodopa, their most frequent single dose of IR levodopa should be selected to determine the recommended starting dosage of IPX203 (Hopledo).

(^a^) For patients where two or more immediate-release levodopa doses correspond to the most frequent immediate-release levodopa doses, the suggested Hopledo (IPX203) conversion should be dosed based on the higher of the immediate-release levodopa/carbidopa doses.

(^b^) For patients whose most frequent immediate-release levodopa dose is 200 mg or higher, clinicians may consider initiating Hopledo (IPX203) using the next lower available capsule strength (70 mg less levodopa per dose) than that derived from the strict 2.8-fold conversion.

After the initial conversion, patient follow-up after 1 to 3 days is recommended, with subsequent adjustment of the dose or dosing frequency based on the individual patient’s response to achieve the optimal balance of efficacy and tolerability. Clinical experience with total daily Hopledo (IPX203) doses above 2100 mg is limited; therefore, care should be taken when considering further dose increases and, in any case, the maximum daily doses of 2380 mg/595 mg should not be exceeded. The total daily dose can be divided up to four doses per day.

IR, immediate-release

# References

1.	European Medicines Agency. Hopledo. Summary of product characteristics. 2026. <https://www.ema.europa.eu/en/documents/product-information/hopledo-epar-product-information_en.pdf>. Accessed 01 Sept 2026
